# EMD-57033 Augments the Contractility in Porcine Myocardium by Promoting the Activation of Myosin in Thick Filaments

**DOI:** 10.3390/ijms232314517

**Published:** 2022-11-22

**Authors:** Vivek Jani, Wenjing Qian, Shengyao Yuan, Thomas Irving, Weikang Ma

**Affiliations:** 1 Department of Biomedical Engineering, The Johns Hopkins School of Medicine, The Johns Hopkins University, Baltimore, MD 20205, USA; 2 Division of Cardiology, Department of Medicine, Johns Hopkins University School of Medicine, Baltimore, MD 21205, USA; 3 Department of Biology, Illinois Institute of Technology, Chicago, IL 60616, USA; 4 BioCAT, Department of Biology, Illinois Institute of Technology, Chicago, IL 60616, USA

**Keywords:** EMD-57033, thick filament activation, X-ray diffraction, super-relaxed state (SRX), porcine myocardium

## Abstract

Sufficient cardiac contractility is necessary to ensure the sufficient cardiac output to provide an adequate end-organ perfusion. Inadequate cardiac output and the diminished perfusion of vital organs from depressed myocardium contractility is a hallmark end-stage of heart failure. There are no available therapeutics that directly target contractile proteins to improve the myocardium contractility and reduce mortality. The purpose of this study is to present a proof of concept to aid in the development of muscle activators (myotropes) for augmenting the contractility in clinical heart failure. Here we use a combination of cardiomyocyte mechanics, the biochemical quantification of the ATP turnover, and small angle X-ray diffraction on a permeabilized porcine myocardium to study the mechanisms of EMD-57033 (EMD) for activating myosin. We show that EMD increases the contractility in a porcine myocardium at submaximal and systolic calcium concentrations. Biochemical assays show that EMD decreases the proportion of myosin heads in the energy sparing super-relaxed (SRX) state under relaxing conditions, which are less likely to interact with actin during contraction. Structural assays show that EMD moves the myosin heads in relaxed muscles from a structurally ordered state close to the thick filament backbone, to a disordered state closer to the actin filament, while simultaneously inducing structural changes in the troponin complex on the actin filament. The dual effects of EMD on activating myosin heads and the troponin complex provides a proof of concept for the use of small molecule muscle activators for augmenting the contractility in heart failure.

## 1. Introduction

More than 26 million patients globally suffer from heart failure, which is characterized as insufficient organ perfusion, central venous congestion, and ultimately end-organ failure, all of which drive morbidity and mortality. Several studies have established that depressed calcium activated tension from skinned muscle preparations correlate with hemodynamic indices of systolic function in end-stage heart failure [1,2,3,4,5,6]. Current therapies for heart failure that improve mortality suppress the neurohormonal signaling thereby reducing the contractility, while inotropic therapies augment the contractility at the cost of increased mortality. Recently, a new class of small molecules, direct muscle activators, known as myotropes, augment the contractility without increasing the all cause-mortality and have emerged as a promising therapeutic approach for systolic heart failure [7].

Muscle contractility, is ultimately determined by the properties of the contractile units (sarcomere), is critical in maintaining organ perfusion. Muscle contraction is driven by the cyclic interactions of the myosin-containing thick filaments with actin-containing thin filaments, powered by the ATP hydrolysis in the sarcomeres. Classically, muscle contraction is thought to be regulated by a calcium-dependent thin filament activation mechanism. Calcium binding to the troponin complexes of thin filaments [8,9] triggers a cascade of conformational changes that lead to the tropomyosin moving away from the myosin binding sites on actin, thereby allowing myosin heads to form force-generating cross-bridges resulting in the sarcomere shortening [10,11]. The binding of myosin to these exposed sites leads to the further activation of the thin filament [10,12,13]. More recently, there is an increasing appreciation that the transitioning of myosin heads from OFF states sequestered on the thick filament backbone to ON states, where the heads are free to interact with actin, is critical in regulating the muscle contractility. An expression for the ensemble force (F_ens_) of the sarcomere can be written as F_ens_ = F_int_·N_a_·t_s_/t_c_, where F_int_ is the intrinsic force of a powerstroke, N_a_ is the number of the functionally accessible heads that binds actin during contraction, and t_s_/t_c_ is the duty ratio, i.e., the portion of the ATPase cycle that the motor domain remains strongly bound to actin [14]. Changes in any combination of these three parameters will affect the ensemble force generated by the sarcomere. A major factor in the hypo- or hyper-contractility in myopathies has been proposed to be the number of functional accessible/inaccessible heads, N_a_ and this concept has been successfully leveraged for depressing the contractility, clinically [14].

The biochemical and structural nature of the functionally accessible and inaccessible myosin heads is an active area of investigation. Using the biochemical quantification of the ATP turnover, myosin can be identified to be either in a state known as the disordered-relaxed (DRX) state with a higher ATP consumption rate (~0.03 s^−1^) or in an energy-sparing state with a low ATP consumption rate (~0.003 s^−1^), known as the super-relaxed (SRX) state [15,16,17]. The ratio of the functionally accessible to the functionally inaccessible heads is believed to be determined by the equilibrium of the myosin heads in the DRX state relative to those in the SRX state [14,18]. Structural measurements using small-angle X-ray fiber diffraction have led to the proposal that the majority of the myosin heads in the resting muscle are quasi-helically ordered on the surface of the thick filament in an OFF state that is not able to interact with actin [19,20,21,22,23]. A small portion of the myosin heads, termed either as constitutively ON [23], or sentinel heads [24], has been proposed to always be in the ON state that are readily available to form cross-bridges. An OFF to ON structural transition has been proposed to underly the transition between functionally inaccessible and accessible heads [25,26]. These biochemical (SRX/DRX) and structural (OFF/ON) definitions of functional accessible/inaccessible heads are generally correlated with each other. For instance, it has been shown in many cases that increases in the population of myosin heads in the SRX state is accompanied by an increase in the ordered OFF heads and vice versa [25,26]. One should keep in mind, however, that the SRX and OFF state heads, and the DRX and ON state heads, are measured in different ways, and do not necessarily correspond to the same underlying phenomena in all cases, so the terms should not be used interchangeably [25,27].

Small molecules directly targeting the sarcomeric proteins have shown promise in regulating the number of functionally accessible heads, N_a_, to correct the contractile dysfunction in cardiomyopathies. However, when it comes to the modes of action of these small molecules, the structural and biochemical estimates of N_a_ are not always in agreement with each other (see above). A desirable drug would correct both the structural and biochemical determinants of N_a_, independent of the inotropic stimulation of the beta-adrenergic pathway, since it is not established which of the two is the most important factor in determining the contractility under a given physiological or pathological condition. Mavacamten, a small molecule direct myosin inhibitor, is the first FDA-approved drug to treat obstructive HCM. Mavacamten is able to promote more heads into the biochemically defined SRX state while shifting them into a structurally defined helically ordered OFF state [26,28]. No other small molecule myosin modulators, besides deoxyadenosine triphosphate (dATP), a precursor of DNA synthesis, have shown these dual effects so far. Thus, the discovery of new compounds with appropriate modes of action would be of significance.

EMD-57033 (EMD) is a thiadiazinone derivative first identified and characterized by Solaro et al. as a thin filament-based calcium sensitizer [29]. Subsequent studies showed that EMD could bind to the C-terminal domain of cardiac troponin C (cTnC) and disrupts the interaction of cardiac troponin between I (cTnI) and cTnC, which might underlie the Ca^2+^ sensitizing mechanism of EMD [30,31]. Surprisingly, a later study by Radke et al. showed that EMD also binds to an allosteric pocket in the myosin motor domain with a one-to-one stoichiometry [32]. The molecular consequences of EMD binding to myosin, however, are not yet understood. Here, using a combination of mechanical (force), biochemical (SRX/DRX), and structural (small angle X-ray fiber diffraction) assays, we showed that EMD decreases the proportion of myosin heads in the biochemically-defined SRX state while moving the heads from a structurally ordered OFF state close to the thick filament backbone to a disordered, ON state closer to actin filament. Establishing that EMD enhances both SRX/DRX and the structural OFF to ON transitions in the myosin heads, but also activates the troponin complex on the thin filament, provides a proof of concept that can guide the search for small molecule muscle activators for correcting the decline in contractile functions associated with heart failure.

## 2. Results

### 2.1. Contractility of Muscle Tissue after the EMD Treatment

**Figure 1:**
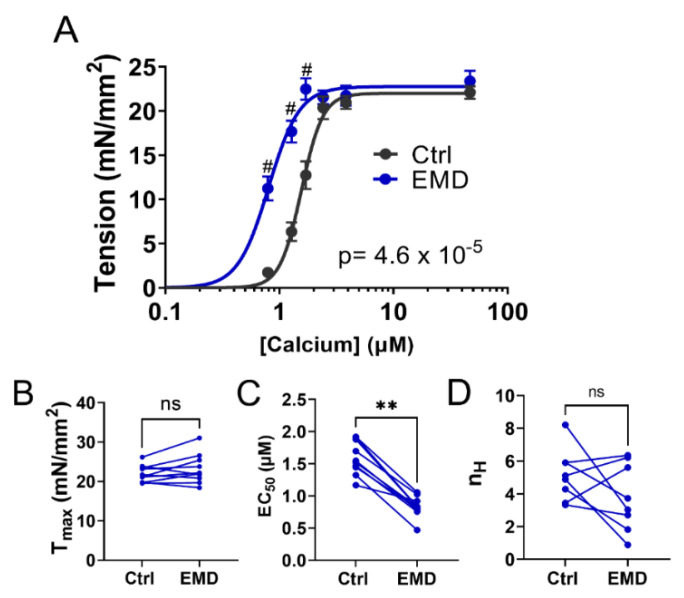
Permeabilized porcine myocardium mechanics with EMD treatment. (**A**) Active tension in a function of calcium concentration in the absence (black) and presence (blue) of 10 uM EMD (EMD). Tension under the maximally activated (T_max_) condition (**B**), the concentration of calcium to achieve the half-maximal activation (EC 50) (**C**) and Hill coefficient (n_H_) of the tension calcium relationship (**D**) before and after the EMD treatment. ns: not significant, ** *p* < 0.01. # *p* < 0.000001.

The permeabilized porcine muscle preparations show a classic sigmoidal tension vs. -log calcium concentration (pCa) relationship (Figure 1A). The addition of EMD induces a left-upward shift in the tension/pCa relationship without significantly increasing the maximal tension (T_max_ = 21.98 ± 0.68 mN/mm^2^ in Ctrl; T_max_ = 23.16 ± 1.17 mN/mm^2^ in EMD; *p* = 0.19; Figure 1B). Tensions at the submaximal calcium concentrations (0.79 μM, 1.28 μM, 1.71 μM) are significantly higher (*p* < 0.000001) post EMD treatment. The left-upward shift of the tension vs. the pCa relationship after the EMD treatment, results in a decrease of EC_50_, the concentration of calcium to achieve the half-maximal activation, (1.59 ± 0.08 μM in Ctrl; 0.83 ± 0.05 μM in EMD; *p* = 0.002; Figure 1C) without significantly changing the Hill coefficient (5.14 ± 0.56 in Ctrl; 3.79 ± 0.73 in EMD; *p* = 0.20; Figure 1D).

### 2.2. Alterations in the Biochemical States of the Myosin Heads with EMD Treatment

**Figure 2:**
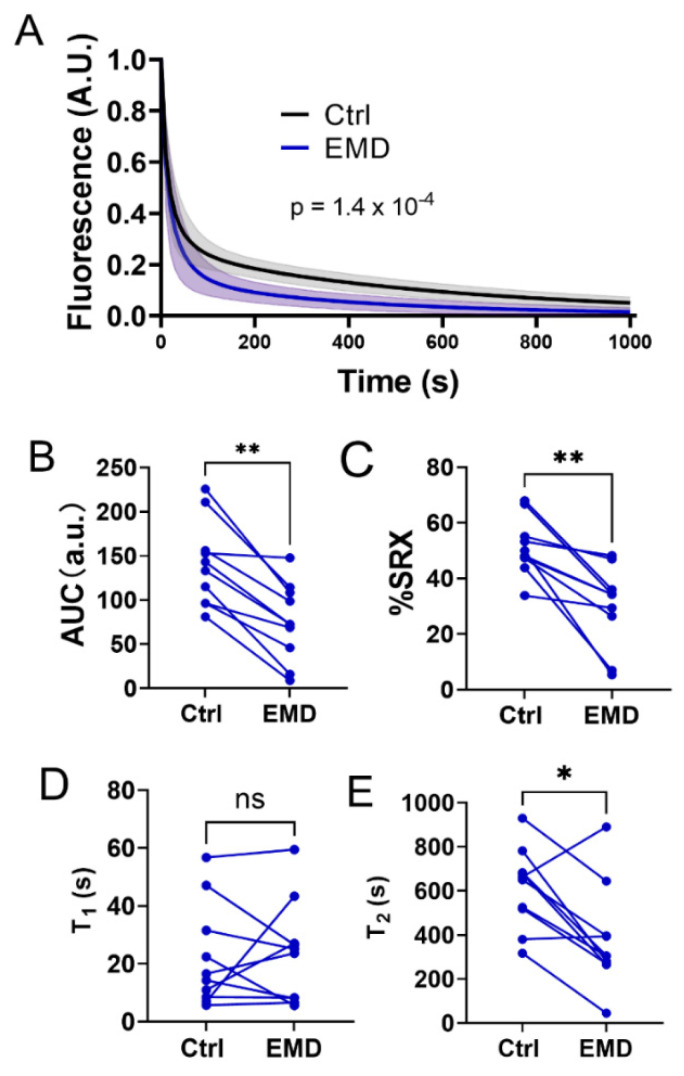
Mant-ATP assay from the permeabilized porcine myocardium before and after the EMD treatment. (**A**) Time course of the mant-ATP dissociation over time curves in the absence (black) and presence (blue) of 10 uM. (**B**) Area under the curve (AUC) of the mant-ATP dissociation time course (through 1000 s) before and after the EMD treatment. (**C**) The percentage of myosin heads in the SRX state (% SRX) before and after the EMD treatment. (**D**) The time constant of the fast phase (T1) before and after the EMD treatment. (**E**): The time constant of the slow phase (T2) before and after the EMD treatment. ns: not significant, * *p* < 0.05, ** *p* < 0.01.

The biochemical states of the myosin heads under relaxed conditions were assessed by a mant-ATP turnover assay before and after the EMD treatment. Upon the EMD treatment, there was a downward shift in the mant-ATP turnover curve (Figure 2A), resulting in a significant decrease in the area under the curve (AUC) (141.1 ± 15.2 vs. 75.45 ± 13.9, Ctrl vs. EMD respectively, Figure 2B, *p* = 0.002), indicating an increased in the ensemble ATPase activity under relaxing conditions in the presence of EMD. The percentage of myosin heads in the SRX state was calculated by fitting the fluorescence decay signals with a two-phase exponential decay. EMD significantly decreased the percentage of the myosin heads in the SRX state from 51.3 ± 3.2% to 30.19 ± 4.5% (Figure 2C, *p* = 0.002). The time constant of the fast phase (T1) and the slow phase (T2) was also calculated. There were no significant changes in T1, post EMD treatment, while T2 is significantly decreased in the presence of EMD (612.8 ± 57.8 s vs. 379.8 ± 73.7, Ctrl vs. EMD respectively, Figure 2D, *p* = 0.01).

### 2.3. Changes in the X-ray Equatorial Diffraction Patterns with EMD

**Figure 3:**
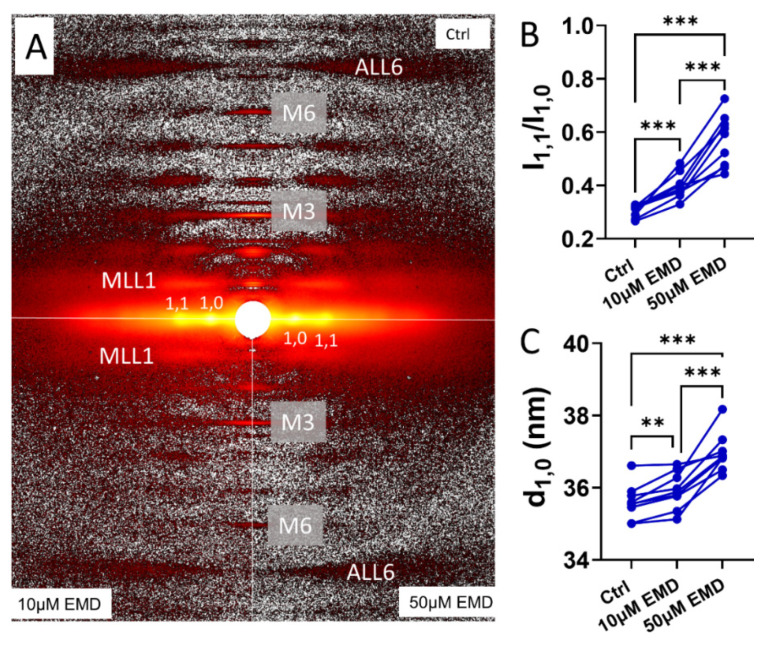
Effects of EMD on the equatorial reflections on the permeabilized pig myocardium. (**A**) Two dimensional diffraction pattern from the permeabilized resting pig myocardium in the absence (Ctrl, top half) and presence of 10 μM (bottom left) and 50 μM (bottom right) ,of EMD. (**B**) Equatorial intensity ratio from the permeabilized pig myocardium in the absence and presence of 10 μM and 50 μM EMD. (**C**) Lattice spacing from the permeabilized pig myocardium in the absence and presence of 10 μM and 50 μM EMD. ** *p* < 0.01, *** *p* < 0.001.

The permeabilized porcine myocardium in the relaxing solution produced the characteristic resting X-ray diffraction patterns in the absence of EMD (top panel of Figure 3A), similar to those reported previously [26]. The hexagonally packed myofilaments inside the sarcomere give rise to the equatorial reflections. The ratio of the intensity of the 1,1 equatorial reflection to that of the 1,0 equatorial reflection, I_1,1_/I_1,0_, is an indicator of the proximity of the myosin heads to actin [33,34] in the relaxed state. I_1,1_/I_1,0_ increased from 0.30 ± 0.007 to 0.40 ± 0.02 (*p* = 0.0007) when the muscles were treated by 10 μM EMD, and it increased further to 0.57 ± 0.03 (Figure 3B) when the EMD concentration was increased to 50 μM at pCa8. The increase of I_1,1_/I_1,0_ indicates a shift of mass, in the form of the myosin heads, away from the thick filament backbone towards the actin containing thin filaments, in the presence of EMD. The interfilament lattice spacing, d_1,0_, increased from 35.6 ± 0.16 nm to 35.9 ± 0.17 nm after the treatment by 10 μM EDM (*p* = 0.005) and increased further to 37.0 ± 0.18 nm in 50 μM EMD (*p* < 0.005) (Figure 3C).

### 2.4. Changes in the Meridional X-ray Reflections and the Layer Lines with EMD

**Figure 4:**
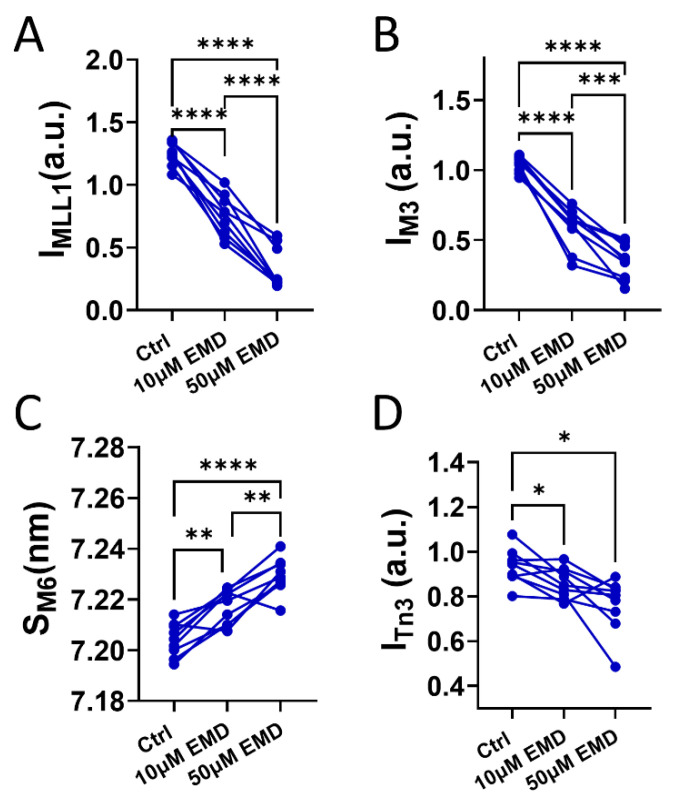
Effects of EMD on the myosin layer lines and the meridional reflections on the permeabilized pig myocardium. (**A**) Intensity of the first order myosin-based layer lines (I_MLL1_) from the permeabilized resting pig myocardium in the absence and presence of 10 μM and 50 μM EMD. (**B**) Intensity of the third order myosin meridional reflections (I_M3_) in the absence and presence of 10 μM and 50 μM EMD. (**C**) Spacing of the sixth order myosin meridional (S_M6_) in the presence and absence of 10 μM and 50 μM EMD. (**D**) Intensity of the third order troponin meridional reflections (I_Tn3_) in the presence and absence of 10 μM and 50 μM EMD. * *p* < 0.05, ** *p* < 0.01, *** *p* < 0.001, **** *p* < 0.0001.

Qualitatively, the intensity of the myosin-based reflections, notably that of the third order myosin meridional reflection, I_M3_, and that of the first myosin layer line, I_MLL1_, reported here, become weaker as the EMD concentration increases (bottom panel of Figure 3A), while the intensity of the sixth order actin-based layer line (I_ALL6_) remains relatively stable. Under resting conditions, the majority of the myosin heads are quasi-helically ordered on the surface of the thick filament with a higher diffraction intensity being correlated with better ordering. These helically ordered myosin heads are structurally defined as OFF heads that are less likely to interact with actin. These OFF heads need to be turned ON to participate in the contraction. The structural ON state is characterized by an increase in the thick filament periodicity, as evidenced by the increases of the M6 meridional reflection spacing (S_M6_) and a reduction in the degree of the helical ordering of the myosin heads, characteristic of the OFF state. The intensity of the first-order myosin-based layer line (I_MLL1_) and the third-order myosin-based meridional reflection (I_M3_), both of which correlate with the ordering of the myosin heads [25,35], decreases when the myosin heads lose their helical ordering. I_MLL1_ decrease from 1.25 ± 0.03 in the control to 0.76 ± 0.05 when the muscles were treated by 10 μM EMD, and further decreased to 0.33 ± 0.06 at 50 μM EMD at pCa8 (Figure 4A). Compared to the control, I_M3,_ decreased from 1.04 ± 0.02 to 0.59 ± 0.05 and 0.35 ± 0.04 at 10 μM and 50 μM EMD, respectively (Figure 4B). The intensity of the sixth-order myosin-based meridional reflection (M6) arises primarily from the structures within the thick filament backbone, so that the spacing of the M6 reflection (S_M6_) reports the periodicity of the thick filament backbone [25]. Increases in S_M6_ have been proposed as a signature of an OFF (inactive myosin heads) -to-ON (active myosin heads competent to bind actin) transition in the thick filament strain-dependent activation mechanism [23]. S_M6_ increases from 7.20 ± 0.002 nm in the control group to 7.22 ± 0.002 nm and 7.23 ± 0.002 nm at 10 μM and 50 μM EMD, respectively (Figure 4C). The troponin complex arranged on the surface of the thin filament at ~37 nm axial spacing gives rise to the troponin based meridional reflections. The intensity of the third order troponin reflection (I_Tn3_) decreased from 0.93 ± 0.03 to 0.86 ± 0.02 and 0.76 ± 0.04 at 10 μM and 50 μM EMD, respectively (Figure 4D).

## 3. Discussion

### 3.1. EMD Recruits Myosin from the Biochemically-Defined SRX State

From a biochemical perspective, myosin is an ATPase that converts ATP to ADP and inorganic phosphate (Pi), thereby converting the chemical energy stored in ATP to mechanical energy. Under relaxing conditions, myosin can adopt different biochemically defined states with different ATPase activities, most notably the SRX and DRX states, the relative proportions of which are proposed to determine the contractility of the muscle during contraction [14,18,36]. Our preliminary studies showed that an increase in the population of myosin heads in the SRX state, might be an underlying cause of a depressed myocardial contractility seen in a cohort of right heart failure patients with group 2 pulmonary hypertension [37]. These preliminary findings indicate that the components of the contractile machinery in systolic heart failure patients are not lost, but rather are merely sequestered in an inactive state and further suggest that recruiting these SRX heads may be a viable mechanism to restore the contractility in end-stage systolic HF. Our data presented here indicate that the EMD treatment increases the ensemble ATPase activity of the permeabilized porcine myocardium. Radke et al. [32] showed that EMD increases the ATPase activity in S1 porcine cardiac myosin, while Solaro et al. [29] showed that EMD did not change the ATPase activity in isolated canine cardiac myosin. The reasons for these discrepancies are not clear. It seems highly unlikely that this is a species-related issue, since Senzaki et al. [38] showed that EMD significantly enhanced the canine myocardium contractility in vivo. Our mant-ATP assays also revealed that EMD is able to recruit the myosin heads from the SRX pool to the DRX pool (Figure 2). This increase in the fraction of the functionally available DRX heads, together with EMD’s calcium sensitizing capabilities, may contribute to the force augmentation seen in Figure 1. It is worth noting that EMD significantly decreases the time constant of the slow phase (T1) of the SRX heads without significantly changing the time constant of the fast phase (T2) of the DRX heads. These results indicate that EMD recruit the SRX heads by altering the ATP turnover rate in the SRX population of the heads, in addition to simply increasing the population of the DRX heads, accelerating the overall ensemble ATPase activity.

### 3.2. EMD Recruits Myosin from the Structurally-Defined OFF State

Structurally, under relaxing conditions, the majority of the myosin heads are arranged in a quasi-helically ordered OFF state on the surface of the thick filament and are less likely to be able to interact with actin and generate force. These quasi-helically ordered myosin heads are assumed to be the structural basis of the SRX state of myosin under physiological conditions but this may not always be the case [25]. Here, we show that EMD was able to promote the myosin heads from the structurally-defined OFF state to the ON state. Specifically, in the presence of EMD, the myosin heads move away from the thick filament backbone toward the actin filaments (Figure 3B), facilitating the cross-bridge formation and increase contractility. The interfilament lattice spacing, d_1,0_, also increases in the presence of EMD. This increase of lattice spacing could be a result of the increased electrostatic repulsion between the myofilaments when the myosin heads move away from the thick filament backbone towards the actin filaments [39,40]. This notion is supported by the previous studies where d_1,0_ is decreased when the myosin heads move towards the thick filament backbone in the presence of the myosin inhibitors, i.e., mavacamten [26]. In the presence of EMD, the myosin heads lose their helical ordering, as indicated by a decrease in I_MLL1_ and I_M3_ (Figure 4A,B). These disordered ON heads observed under relaxing conditions, are generally believed to be equivalent to the biochemically defined DRX heads but this may not always be the case [25].

### 3.3. Sarcomeric Activators as an Approach for Rescuing the Contractility in Myocardium

Depression of the myocardial contractility, independent of the ejection fraction, is commonly seen in failing hearts [1,2,3,4,5,41]. Current inotropic therapies that directly augment the contractility are problematic, clinically, as they are pro-arrhythmic and increase mortality and, as such, are restricted to acute decompensation. Increasing the myocardium contractility by the direct sarcomeric activation, therefore, may be a straightforward solution to correct the depressed contractility. EMD is the first identified activator that acts directly on a sarcomeric protein (troponin) [29] and has been shown to increase myocardium contractility in various systems [38,42,43]. Consistent with previous studies, we show here that EMD increases the calcium sensitivity (decrease of EC_50_) thereby increasing the myocyte contractility at the physiological calcium concentrations. Calcium sensitivity is an important parameter in assessing the muscle function. Traditionally a calcium sensitivity has been considered primarily a property of the thin filaments, since the troponin complex is the primary calcium sensor [44,45], followed by the formation of the strong binding crossbridges that further activate the thin filament [13]. Calcium sensitivity may also be altered by post translational modifications of the sarcomeric proteins [46]. In this study, we show that EMD shifts the myosin heads towards the thin filament with a higher ATPase activity and these structural changes in the thick filament may be an important component of the overall calcium sensitivity of the sarcomere. The close proximity of the myosin heads to actin would be expected to facilitate the formation of the crossbridges once the thin filament is turned on by the initial calcium binding. The formation of the strong binding crossbridges will further activate the thin filament, which could be, at least partially, responsible for the increased calcium sensitivity, post EMD treatment. One recent study indicated that the myosin filaments can be directly regulated by calcium [47]. Calcium can turn the myosin filament ON in the absence of the thin-filament based activation, indicating that the calcium sensitivity might not be a exclusively a thin filament property.

The new focus on the thick filament activation processes suggests new insights into the action of the myosin activators. It is generally assumed that the biochemically-defined SRX/DRX states are correlated with the structurally-defined OFF/ON states under physiological conditions, and both of them are believed to be contribute to muscle’s contractility [18,22,36]. Here we show that EMD affects both kinds of transitions. EMD was first identified as a troponin binding compound that can shift the OFF/ON equilibrium of the thin filament toward the ON state [30,31]. Our data showed that I_Tn3_ decreased in the presence of EMD under relaxing conditions. At this point, there is no intuitive way to interpret the observed decrease in I_Tn3_, besides the binding of EMD on the troponin complex, inducing a structural change in the troponin complex. Along with the previously demonstrated changes in the troponin X-ray reflections with a passive stretch [21], these results suggest that further exploration of the relationship of the changes in the troponin structure, under relaxed conditions to the subsequent contractility, could be a fruitful avenue of inquiry.

EMD-57033 has not been tested in clinical trials, due to issues with its bioavailability [48,49]. EMD-57033 is a lipophilic compound with BCS Class II properties (high permeability, low solubility) [50]. The low solubility of EMD-57033 in aqueous solutions (5 μg/mL at 37 °C) limits its bioavailability [51]. In addition, only intravenous formulations of EMD-57033 can be used for treatment, further limiting its clinical application. The known structure of EMD-57033, however, may aid in the search and development for compounds that have a higher biological availability. Muscle regulation is now understood to be regulated by both thick and thin filament based mechanisms. Since EMD-57033 can activate the myosin heads from both the biochemically-defined SRX and the structurally-defined OFF states, synergistically, with activating the troponin complex, it can provide a proof of concept to guide the search for other small molecule muscle activators with similar properties that can correct the decline in contractile functions associated with heart failure, without significantly impairing the ventricular filling in diastole.

## 4. Materials and Methods

### 4.1. Isometric Tension-Calcium Relationships

Frozen wild type, left ventricular wall myocardium was provided by Exemplar Genetics Inc (Sioux Center, IO, USA). Tension-calcium curves for the skinned cardiomyocytes (CM) were acquired, as described previously [2,6]. Briefly, the frozen porcine myocardium was cut over dry ice into 10–15 μg pieces and incubated in ice-cold (0 °C) skinning solution (isolation buffer: 5.55 mM Na_2_ATP, 7.11 M MgCl_2_, 2 mM EGTA, 108.01 mM KCl, 8.91 KOH, 10 mM Imidazol, 10 mM DTT + 0.3% Triton X-100) with protease (Sigma-Aldrich, St. Louis, MO, USA) and phosphatase inhibitors (PhosSTOP, Roche, Mannheim, Germany). The tissue was homogenized with low-speed pulverization, skinned for 20 min at 4 °C, and washed with isolation buffer. The CMs were attached to a force transducer-length controller (Aurora Scientific, Aurora, ON, Canada) using an ultraviolet-activated adhesive (Norland Optical Adhesive 63, Norland, East Windsor, NJ, USA), moved into room temperature (~22 °C) relaxing buffer (5.95 mM Na_2_ATP, 6.41 mM MgCl_2_, 10 mM EGTA, 100 mM BES, 10 mM CrP, 50.25 mM Kpropionate, protease inhibitor (Sigma-Aldrich, St. Louis, MO, USA), 1 mM DTT), and set to a 2.1 μm sarcomere length, as assessed by the fast Fourier transform of images (Aurora Scientific Software, IPX-VGA210, Imperx, Boca Raton, FL, USA).

Tension-Ca^2+^ relationships were acquired by varying the Ca^2+^ concentration from 0.0–46.8 μM. Force was normalized to the cross-sectional area estimated as $\frac{\pi }{4}ab$, where a is the diameter of the myocyte from the camera and b is the short axis diameter approximated as 0.8 a, to obtain tension (mN/mm^2^). The steady-state tension versus the log [Ca^2+^] plots (T-Ca^2+^ plots) were fit to the three-element Hill equation: T = T_max_ × Ca*^nh^*/(EC_50_*^nh^* + Ca*^nh^*), where T_max_ is the maximum calcium-activated tension, EC_50_ is the calcium sensitivity, and n_h_ is the Hill coefficient. The resting tension at a 2.1 μm sarcomere length was subtracted from the total tension measured to obtain the Ca^2+^ activated tension. Following the acquisition of the first tension-Ca^2+^ relationship, the CMs were incubated in the relaxing solution with 10 μM EMD-57033 (EMD) (Millipore Sigma, Burlington, MA, USA) for 10 min, and the tension-Ca^2+^ relation post-exposure was obtained.

### 4.2. Myosin ATP Turnover Kinetics

The proportion of the super-relaxed (SRX) myosin was obtained from the skinned CMs, as described previously [52,53]. The sarcomere length was set to 2.1 μm in a relaxing buffer for all experiments. The cells were affixed to a force and length transducer and washed in rigor buffer (6.41 mM MgCl_2_, 10 mM EGTA, 100 mM BES, 10 mM CrP, 50.25 mM Kpropionate, protease inhibitor (Sigma-Aldrich, St. Louis, MO, USA), 10 mM DTT) to remove ATP and was subsequently incubated in the relaxing buffer made with 25 μM 2′-/3′-O-(N’-Methylanthraniloyl) adenosine-5′-O-triphosphate (mant-ATP, Enzo Life Sciences, Axxora LLC, Framingham, NY, USA). The CMs were moved to the relaxing buffer and the fluorescence acquired (excitation 352–402 nm, emission 417–444 nm; Horiba/PTI 814 Photomultiplier Detection System), continuously at 100 Hz for 1000 s. The acquired fluorescence signal was filtered using a second order Savitzky–Golay filter and was normalized and fit to a biexponential function, I = 1 − P_1_(1 − e^−t/T1^) − P_2_(1 − e^−t/T2^). The proportion of the SRX myosin is 2 × P_2_, and the percentage of the DRX myosin is 1 − (2 × P_2_). The background noise was limited, using the IonOptix Cell Frame Adapter (CFA, Westwood, MA, USA). The background was subtracted by measuring the average photomultiplier tube voltage output in the surrounding relaxing buffer at the end of the assay for each CM. Following the acquisition of the fluorescence decay curve, the CMs were incubated in 10 μM EMD-57033 (Millipore Sigma, Burlington, MA, USA) relaxing solution for 10 min, and the assay was repeated post exposure. All analyses were performed using custom routines written in Matlab (Mathworks, 2018, 2020).

### 4.3. Muscle Preparations for the Small-Angle X-Diffraction

The muscle samples were permeabilized, as described previously [54]. Briefly, the muscles were permeabilized in skinning solution (2.25 mM Na_2_ATP, 3.56 mM MgCl_2_, 7 mM EGTA, 15 mM sodium phosphocreatine, 91.2 mM Potassium Propionate, 20 mM Imidazole, 0.165 mM CaCl_2_, 15 mM 2,3-Butanedione 2-monoxime (BDM), creatine phosphate kinase 15 U/mL and 1% Triton X-100) and the protease inhibitor cocktail for ~30 min before splitting them into smaller fibers. The fibers were transferred into fresh skinning solution and incubated overnight at 4 °C. The muscles were then washed with fresh relaxing solution (pCa8: 2.25 mM Na_2_ATP, 3.56 mM MgCl_2_, 7 mM EGTA, 15 mM sodium phosphocreatine, 91.2 mM Potassium Propionate, 20 mM Imidazole, 0.165 mM CaCl_2_) three times, 10 min each, to wash out the BDM and Triton X-100. The muscles were further dissected into fiber strips ~200 μm diameter and clipped with aluminum T-clips and subsequently stored in the cold relaxing solution at 4 °C for the day’s experiments. The X-ray diffraction patterns were collected using the small-angle instrument on the BioCAT beamline 18ID at the Advanced Photon Source, Argonne National Laboratory [55], as described previously [54]. The diffraction patterns were collected at sarcomere lengths of 2.3 μm in the absence and presence of 10 μM and 50 μM EMD. The X-ray patterns were collected on a CCD-based X-ray detector (Mar 165; Rayonix Inc. Evanston, IL, USA) with a 1 s exposure time at an incident flux of ~3 × 10^12^ photons per second under each condition. The equatorial X-ray diffraction patterns were analyzed using the Equator module from the MuscleX (version 14.2) software package developed at BioCAT [56], as described [34]. The X-ray patterns were subsequently quadrant folded and the background was subtracted to improve the signal to noise ratio, for further analysis using the Quadrant Fold module of the MuscleX program suite. The meridional and layer line reflections were measured using the Projection Traces module of the MuscleX program suite, as described [26,57]. Three to four patterns were collected under each condition and the X-ray reflection data extracted from these patterns were averaged.

### 4.4. Statistics

The statistical analyses were performed using GraphPad Prism 9 (Graphpad Software. version 9.4.1). The results are given as mean ± SEM. Data shown in Figure 1A were fit with a specific binding with a Hill function and the individual tension levels pre- and post- EMD treatment were compared by a 2 way ANOVA with Šídák’s multiple comparisons test. Data shown in Figure 1A were compared using a 2-way repeated measures ANOVA. The remainder of the panels in Figure 1 and Figure 2 were analyzed using a Wilcoxin matched-pairs *t* test. Data shown in Figure 3 and Figure 4 were analyzed using the repeated measure, one-way ANOVA with Tukey’s multiple comparisons test. Symbols on the figures: ns: *p* >= 0.05, *: *p* < 0.05, **: *p* < 0.01, ***: *p* < 0.001 and ****: *p* < 0.0001, #: *p* < 0.000001.

## Data Availability

All datasets generated for this study are included in this article. The raw data are available from the corresponding author (Weikang Ma: wma6@iit.edu) upon reasonable request.
